# Salt restriction in kidney disease—a missed therapeutic opportunity?

**DOI:** 10.1007/s00467-008-0856-4

**Published:** 2009-01-01

**Authors:** Eberhard Ritz, Otto Mehls

**Affiliations:** 1 Department of Internal Medicine, Ruperto Carola University of Heidelberg, Nierenzentrum, Im Neuenheimer Feld 162, 69120 Heidelberg, Germany; 2 Division of Pediatric Nephrology, University Children’s Hospital of Heidelberg, Heidelberg, Germany

**Keywords:** Chronic kidney disease (CKD), Salt intake, Salt restriction, Programming of blood pressure, Hypertension, Target organ damage, Cardiotonic steroids

## Abstract

The importance of salt restriction in the treatment of patients with renal disease has remained highly controversial. In the following we marshal the current evidence that salt plays a definite role in the genesis of hypertension and target organ damage, point to practical problems of salt restriction, and report on novel pathomechanisms of how salt affects blood pressure and causes target organ damage.

## Salt and blood pressure

### Adults

In a crucial experiment, Denton et al. [1] measured the blood pressure of chimpanzees on a salt-enriched diet and control chimpanzees on a no-added salt diet. After 6 months in chimpanzees on a salt enriched diet blood pressure had increased in chimpanzees on a salt-enriched diet, from 110 mmHg systolic to 145 mmHg, while it had not changed in the control group. The effect was fully reversible after the high salt diet was stopped. Meanwhile, the Dietary Approaches to Stop Hypertension (DASH) study provided convincing evidence that a diet with reduced salt (NaCl) intake [but also additional dietary manipulation, particularly higher potassium (K) intake] decreased systolic blood pressure: when sodium (Na) intake was reduced from 150 mmol/day to 50 mmol/day, systolic blood pressure (office blood pressure) decreased by 6.7 mmHg in the overall cohort, and, in hypertensive individuals, even by 11 mmHg [2]. This may even have been an underestimate. Using the more sensitive ambulatory blood pressure measurement, a double-blind placebo-controlled crossover trial recently showed that reduction of salt intake by 50 mmol/day from 150 mmol/day to 100 mmol/day lowered 24 h blood pressure by no less than 5.7 mmHg systolic and by 6.3 mmHg at night time [3].

The change in blood pressure with salt restriction is particularly marked in patients with renal failure [4], and this parallels the observation of an exaggerated blood pressure increase in animals on a high salt intake that was characterized by reduced numbers of nephrons [5].

### Children, programming and salt sensitivity of blood pressure

More than 20 observational studies on salt and blood pressure in children were reviewed by Simons-Morton and Obarzanek in 1997 [6]. The majority of studies showed a significant positive association between salt intake and blood pressure. In the study by Cooper and Liu [7] a significant linear relationship between urinary sodium and blood pressure in 73 children aged 11 years to 14 years was noticed after the authors had controlled for age, gender, race, pulse rate, height and body weight. The same result was noticed in 233 Dutch children aged 5 years to 17 years and followed up for ≥ 7 years [8]. A meta-analysis of ten controlled trials with 966 children and adolescents was reported by He and MacGregor [9]. Mean salt intake was reduced by 42%, and the median duration was 4 weeks, ranging from 2 weeks to 3 years. Both systolic (−1.17 mmHg) and diastolic (−1.29 mmHg) blood pressures were significantly decreased. In three trials on infants, including 541 participants, salt intake was reduced by 54% and the systolic blood pressure by 2.47 mm Hg. The authors concluded that neonatal salt intake may induce blood pressure tracking and late hypertension.

Many experimental studies have documented that the fetal environment, including salt intake of mothers, can have long-lasting effects on blood pressure, but salt intake was not studied as an independent factor.

Exposure of mothers to low or high salt throughout gestation had a long-lasting impact on salt preference of offspring [10, 11]. In the study by Ross et al. lambs were exposed to in utero hypernatremia induced by maternal water restriction [12]. Offspring of prenatally water-restricted mothers exhibited hypernatremia, hypertonicity and hypertension. Thus, maternal nutrient stress may program altered osmoregulation and systemic arterial hypertension in the offspring. Interestingly, Crystal and Bernstein [13] demonstrated that infants of mothers who had suffered morning sickness during early pregnancy drank more concentrated salt solutions than controls. In young students whose mothers reported morning sickness, Málaga et al. also found a greater preference for salty food [14]. In infants and young children, salt taste preference was inversely related to birth weight over the first 4 years of life [15].

Thus, the early response to salty tasting food might predict future sodium intake and blood pressure.

Porter et al. [16] were the first to investigate whether high salt intake in the mother programmed blood pressure and heart rate hyper-responsiveness to stress in adult offspring independently of birth weight. A high sodium diet administered to pregnant rats during the perinatal period was not sufficient to program lasting hypertension (measured by telemetry) or salt sensitivity of blood pressure in offspring. However, enhanced pressure and tachycardiac response to acute stress persisted into adulthood. This was accompanied by increased mRNA expression of corticotrophin-releasing hormone under basal and stress conditions. The programmed defects did not depend on birth weight and might have involved other mechanisms, for instance augmented activation of the sympathetic nervous system.

According to the Borst–Gyton concept, chronic hypertension occurs only with a shift in the renal pressure–natriuresis relationship, resulting in increased salt sensitivity of blood pressure. Birth weight was negatively associated with salt sensitivity of blood pressure, and this was independent of the association between birth weight and glomerular filtration rate (GFR) [17]. Kidney and central nervous system seem to be the major sites for salt sensing: by chloride sensing in renal tubule fluids via sodium–potassium–chloride (Na-K-Cl) co-transporter isoforms in the macula densa cells; by sodium sensing in cerebrospinal fluid; by the sensing of cerebrospinal fluid osmolarity by mechanosensitive, non-selected cation channels, and by osmolarity sensing in glial cells [18]. The plurality of salt-sensing mechanisms allows very precise control of blood pressure. Modifications of sensor programming or of feedback signaling are strong candidates for the programming of blood pressure and salt sensitivity. Other options for the development of salt-sensitive hypertension are genetic and congenital factors, which might predispose individuals to microvascular disease; interstitial damage; local angiotensin II formation, and oxidant generation [19].

### Effects of salt intake on cardiovascular and other endpoints

Apart from the effect of salt intake on blood pressure, its effect on cardiovascular endpoints is of interest as well. A recent meta-analysis showed by extrapolation that, in the general population, reduction of salt intake by 3 g/day, and the resulting decrease in blood pressure, would translate into a 13% reduction in stroke and a 10% reduction in ischemic heart disease events [9]. An observational study from Finland even suggested that a 100 mmol difference in salt intake would reduce the relative risk of coronary heart disease by 50%, independent of blood pressure [20].

Apart from these observational studies, there is only one series of two small prospective but long-term (10–15 years) studies available in which salt intake was reduced by 44 mmol/day and 33 mmol/day, respectively. These Trials of Hypertension Prevention (TOPH) I and TOPH II studies document a significant impact of moderate salt reduction on cardiovascular endpoints by 25%. There was even a trend for reduction of mortality by 20% [21].

Apart from the information on cardiovascular events, limited information is also available on the effect of high salt intake on renal parameters. Salt load increases the local concentration of angiotensin II in the kidney, thus constricting the efferent vessels of the glomeruli followed by an increase in intraglomerular pressure and filtration [19]. Verhave et al. [22] observed that urine albumin excretion in the general population was correlated with the magnitude of sodium excretion; this correlation was most marked in individuals in the highest tertile of the body mass index. A correlation between salt intake and albuminuria has also been found in the rat uninephrectomy model [23] and in the Munich Wistar rat with spontaneously reduced nephron numbers [5]. Albumin excretion rates were also higher in rats with intra-uterine growth retardation [24].

Of considerable interest are observations on the effect of salt intake in rats with renal injury. Dworkin et al. [25] kept subtotally nephrectomized rats for 4 weeks on standard chow and subsequently randomly allocated them either to low salt chow or standard chow. A reduction in proteinuria was observed in the rats on the low-salt chow and progression in rats on standard chow; this was accompanied by a further increase in the proportion of sclerotic glomeruli in the latter group.

Salt loading also promotes renal fibrosis in numerous, but not all, models of renal damage [26, 27]. Of particular interest is the observation that the effect of salt restriction cannot be reproduced by the administration of a diuretic [28].

## Mechanisms of salt-induced target organ damage

In patients with chronic kidney disease a high salt intake may be “nephrotoxic” by indirect mechanisms, e.g. by an increase in blood pressure and by attenuation of the pharmacologic blockade of the renin–angiotensin system blunting its anti-hypertensive and anti-proteinuric effect. The latter was elegantly documented when patients with nephrotic proteinuria were treated with lisinopril: the anti-proteinuric effect was markedly reduced by a high-salt diet [29]. In patients with chronic kidney disease, salt loading increases renovascular resistance, with the attendant decrease in renal plasma flow, and increases glomerular capillary pressure as extrapolated from the increase in filtration fraction [30], effects certainly undesirable in patients with chronic kidney disease.

One main pathomechanism underlying salt-induced tissue damage is oxidative stress, a phylogenetically ancient reaction: when plants, such as marigold, grow in an environment with high salinity, oxidative stress occurs and leads to stunted growth [31]. Similarly, high salt intake increases oxidative stress in the mammalian kidney by a combination of increased generation and decreased breakdown of reactive oxygen species (ROS) [32]: the expression of reduced nicotinamide adenine dinucleotide phosphate [NADPH] oxidase mRNA is increased 2–3 fold, whilst the expression of several isoforms of superoxide dismutase is reduced. Increased oxidative stress in the animals on high-salt diets is proven by the decreased urinary excretion of 8-isoprostane, a lipid marker of oxidative stress [32].

High salt intake presumably causes hypervolemia, increases blood flow and shear stress in central arterial vessels and induces expression of endothelial nitric oxide (NO) synthase (eNOS) [33]. In the kidney as well, Sato et al. [34] documented that expression of eNOS mRNA is modulated by salt intake. In the Thy1 glomerulonephritis model this was paralleled by changes in the expression of transforming growth factor beta (TGF-β) [35].

## The right balance between too little and too much salt

Currently there is an animated discussion as to whether reduction of salt intake might have negative consequences. It goes without saying that radical salt restriction causes hypovolemia, with the attendant adverse effects, e.g. reduced resistance against circulatory shock and possibly also predisposition to acute renal failure. Extremely low salt intake also causes more long-term adverse effects, such as acceleration of atherosclerosis, as shown in a genetic model of atherosclerosis, the apoE−/−mouse [36]: a low-salt diet stimulated the renin–angiotensin system and increased the size of atherosclerotic plaques in the aorta. It is also of interest that, in pregnant rats, an extremely low salt intake (0.03%) causes intrauterine growth restriction and adult hypertension in the offspring [37].

**Fig. 1:**
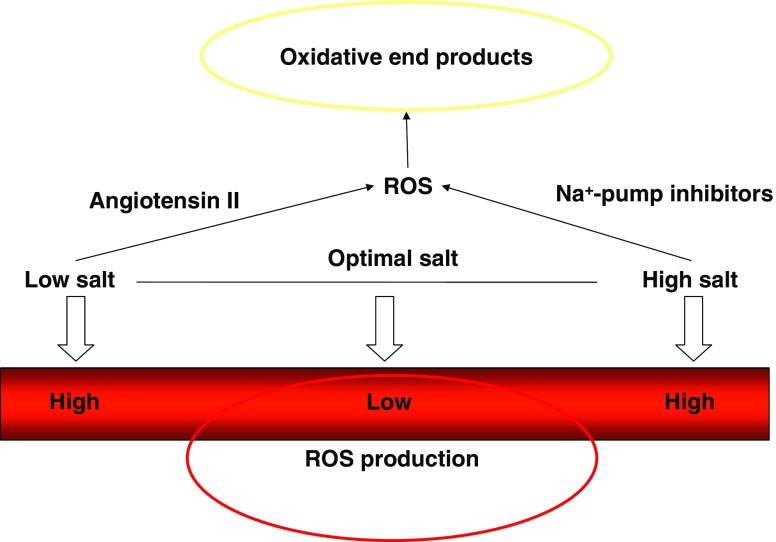
Both too high and too low a salt intake produce ROS and oxidative end products

The explanation of why both too high and too low a salt intake cause adverse effects is the fact that high salt intake induces inhibitors of the Na^+^K^+^ pump (cardiotonic steroids) on the one hand [38], whilst low salt intake up-regulates the activity of the renin–angiotensin system, increases angiotensin II, and up-regulates NADPH oxidase, thus generating reactive oxidant species [39] (Fig. 1).

A sound balance between these two extremes is struck by current guidelines [40], which recommend for adults a reduction in the usual daily sodium intake to 80–100 mmol, corresponding to an intake of 4.7–5.8 g salt.

## What evidence is available in children?

In children, two controlled trials were performed in free-living individuals in whom urinary sodium output was measured. Children with a mean age of 11 years had a mean intake of 133 mmol sodium per day (7.8 g salt) [41]. In 13-year-old boys the mean intake was 150 mmol per day, and in girls it was 142 mmol per day (corresponding to 8.8 g and 8.4 g salt, respectively) [42]. In 4–6 year-old children the average urinary sodium was 64 mmol per day, corresponding to 3.8 g salt [43]. The National Diet and Nutrition Survey for young people, which was carried out in Great Britain in 1997, investigated 1,658 participants aged between 4 years and 18 years regarding their salt intake and blood pressure. The mean salt intake was 4.7 g/day at the age of 4 years, and it increased with age to 6.8 g/day at the age of 18 years. After adjustment for age, gender, body mass index and dietary potassium, an increase of 1 g salt per day was related to an increase of 0.4 mmHg systolic pressure and 0.6 mmHg pulse pressure [44]. In children the proportion of food that children consumed in restaurants and food outlets increased by 300% between 1977 and 1996 [45]. In the Finish Special Turku Coronary Risk Factor Intervention Project (STRIP), children had exceeded the estimated requirements of the US National Academy of Science by about 700% at 13 months, 3 years and 5 years of age [46].

For children no data exist on the physiological ranges required for sodium homeostasis. Therefore, the recommendations for sodium intake in infants are based on the upper end of the breast milk reference intake range, whereas, for older children, the recommendations are estimated factorially by calculation of daily increments in total body sodium content, allowing for changing body composition and compartmentation, such as the declining proportion with age of extracellular fluid in body mass and for dermal, fecal and some urinary losses. According to the dietary recommendations of the Scientific Advisory Committee on Nutrition, the estimated minimum requirements of sodium for 1-year-old children is 225 mg sodium/day (0.6 g NaCl), and for 2–5 year-old children it is 300 mg/day (0.8 g NaCl) [47]. The British reference nutrient intakes (RNIs) for sodium and target average salt intake are given in Table 1.These values have been estimated on the same basis used to derive the recommended target salt intake for adults, i.e. an increase in the RNI by a factor of 1.5. There is insufficient evidence to define precisely the upper limits of salt intake in relation to cardiovascular risk in children.

**Table 1:** Reference nutrient intakes (RNIs) for sodium and target average salt intake for infants and children

Age (years)	RNI		Target average intake (g/day)
	Sodium mmol/day (mg/day)	salt (g/day)	
0–0.5	8 (184)	0.5	< 1
0.5–1	12 (276)	0.7	1
1–6	26 (598)	1.6	2
7–14	60 (1,380)	3.6	5

An unresolved issue remains about how patients can manage to achieve reduction of their salt intake from the current 12–14 g/day for adults to the above recommended level. Unfortunately, according to Sanchez-Castillo [48], table salt contributes only 10% to the daily dietary intake, and no more than 5% is contributed by salt added during cooking—both sources can be directly controlled by patients. In contrast, 85% of salt is latent, added by the food industry and therefore contained in the food items used for cooking. As a result, the recommended moderate reduction of salt intake is difficult to achieve. The best strategy is a community approach involving health policy actions to motivate the food industry to reduce the salt content of food items. This is in line with the American Public Health Association resolution, which postulates that food manufacturers and restaurants should reduce sodium in the food supply by 50% during the next decade [49]. We realize that this is a hot political issue, which has provoked an intense political debate [50].

It has been suggested that, apart from this political resistance against the reduction of salt intake, latent historical reminiscences may charge the issue emotionally [51].

## Resistance to salt restriction—historical reminiscences

**Fig. 2:**
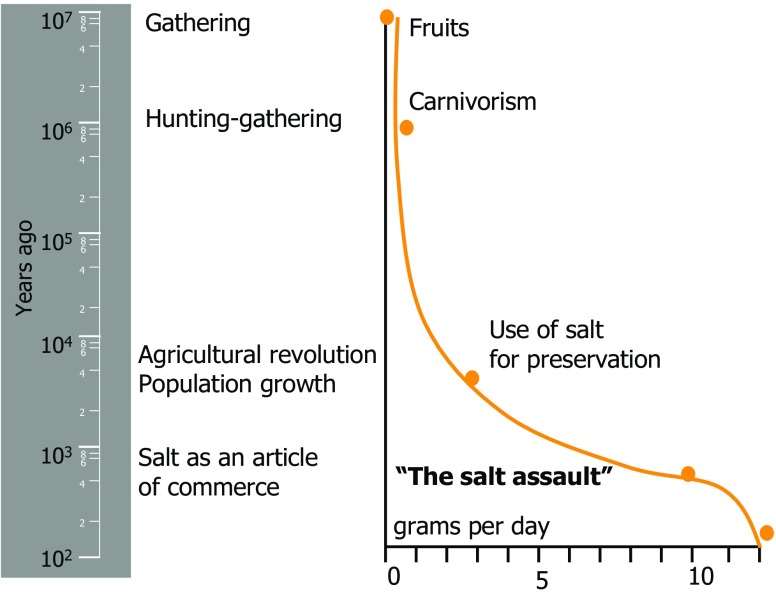
Increase of salt intake during evolution. Courtesy of Dr. P. Ferrari, Department of Nephrology, Fremantle Hospital, Perth, Western Australia, Australia

As shown in Fig. 2, salt intake in the era of hunters and gatherers was very low, presumably less than 1 g per day. It was only during the agricultural revolution and the associated population growth that higher salt intake became a necessity. The current high levels of salt intake, however, were reached only when salt became available as an article of commerce.

As emphasized by several authors, the history of salt as a scarce product of vital importance for survival left presumably historical reminiscences which perpetuate the notion that salt is a valuable essential constituent of the diet [52]. In many cultures around the world, salt, a white substance, is a symbol of the immaculate, the incorruptible, the imperishable, as reflected by the Arabian proverb “Salt is not worm eaten”. It has been an emblem of immortality and a symbol of immutable loyalty, as reflected by the sharing of bread and salt with a guest, a custom still alive in Slavic countries. In the past, ratifying contracts and sealing covenants included the use of salt, as reflected by the quotations in the Bible “It is a covenant of salt forever, before the Lord” (Numbers 18: 19) and “The Lord God of Israel gave the Kingdom over Israel to David forever, even to him and his sons by a covenant of salt” (2 Chronicles 13: 5). Paradoxically from today’s perspective, in ancient Rome salt was even regarded as a symbol of health. This concept is still reflected by the words salus (health) and salubris (healthy) in Latin and the greeting “salut” in French. Officials and soldiers in ancient Rome did not receive money, but a fixed amount of salt (salarium) which is still reflected in the modern words salary (English), salaire (French), sold and soldier (English). Salt had enormous economic value, akin to gasoline today, and this explains why salt production was so important economically that the names of many cities still reflect past salt production: the syllable “wich”, pointing to an ancient saltern, is retained in names such as Sandwich, Ipswich; the Celtic word Hall for salt is still preserved in names such as Hall/Tirol, Halle/Saale; the Germanic words Salz and Salt are still preserved in names such as Salzburg, Langensalza and Saltcotes.

As a final remark, the salt tax was a major cause that sparked the French Revolution. Like the gasoline tax today, salt was an indispensable commodity and, for the authorities, an ideal object for taxation. This invited salt smuggling in pre-revolutionary France, which a special salt police (les gabelous) tried to suppress. They carried out military actions against salt smuggling, searched private property for hidden salt, and, notoriously, molested women (who were, however, frequently hiding smuggled salt in their underwear). The degree of oppression is reflected by the fact that, in the last year before the French Revolution, 3,500 citizens were sentenced to death or the galleys because of salt smuggling. This explains why, in the French Revolution, all salt tax officials were decapitated.

## The importance of salt balance on hemodialysis

When maintenance hemodialysis was introduced by Belding Scribner and colleagues in March 1960, they wrote 6 weeks later in their famous preliminary report [53] “as in the case of nephrectomized dogs, hypertension appears to be influenced by the size of the extracellular space. The combination of dietary sodium restriction and ultrafiltration during dialysis permits the regulation of extracellular volume”. To his last days Scribner emphasized the method of drug-free salt restriction and ultrafiltration to control blood pressure [54]. The high efficiency of a negative salt balance in controlling blood pressure of patients on dialysis was confirmed by numerous others. The original concept that the adverse effect of sodium was mediated exclusively via hypervolemia, as originally suggested by Guyton et al. [55], must, to some extent, be modified, however, in the light of some recent observations by Titze and co-workers [56], who showed that sodium can be stored in the skin and other organs in an osmotically inactive form. It is currently unknown whether this mechanism has repercussions on the blood pressure effect of a positive salt balance in humans. An attractive alternative hypothesis is that salt induced hypertension is the consequence of salt induced cardiotonic steroids (see below).

Obviously, hypervolemia per se does not necessarily raise blood pressure, as exemplified by the observation of low blood pressure in pregnancy, despite hypervolemia. Hypertension occurs only when the vascular resistance is inappropriately elevated (Table 2).

**Table 2:** Causes of inappropriately high vascular resistance in uremia

Cause
Activated renin–angiotensin system
Stimulated sympathetic nerve activity
Reduced breakdown of catecholamines (renalase)
Oxidative stress
Reduced endothelial cell-dependent dilatation (ROS)
Vascular remodeling with structurally fixed elevation of vascular resistance

Only a few centers, e.g. Tassin, in France [57], stuck to a rigorous low-salt diet and use of lower concentrations of sodium in the dialysate as proposed by Scribner. This strategy reduces extracellular volume and total peripheral resistance, yet, it is at the expense of increased norepinephrine and angiotensin II concentrations. Nevertheless, normal blood pressure values are achieved in the vast majority of patients. This is not explained by the prolonged dialysis times at the Tassin center, since similar blood pressure lowering by dietary salt restriction, ultrafiltration and reduction of dialysate sodium was also achieved in patients with dialyses of 5 h duration [58]. By reducing salt intake and by aggressive ultrafiltration, Ozkahya [59] even achieved regression of left ventricular hypertrophy without the use of anti-hypertensive drugs. That such a regime might require blockade of the counter-regulatory increase of the renin–angiotensin system (RAS) (and possibly also of the sympathetic nerve system) was shown by Tycho Vuurmans and colleagues [60]. They studied pulse wave velocity as an index of vascular stiffness: this parameter was not changed by administration of angiotensin-converting enzyme (ACE) inhibitors or by volume removal, yet it was normalized by the combination of the two maneuvers.

One further mechanism for how positive salt balance and hypervolemia may have adverse effects is the ‘intestinal leak’ of endotoxin resulting from edema of the intestinal mucosa. In edematous patients with heart failure Niebauer et al. [61] showed that transit of bacterial lipopolysaccharide across the intestinal mucosa and signs of micro-inflammation were reduced when the edema of the intestinal mucosa was reduced by diuretic treatment.

## Pathomechanisms underlying salt-induced target organ damage: cardiotonic steroids

Ever since Sir William Withering noted the therapeutic efficacy of foxglove in edematous patients with heart disease (“An Account of the Foxglove and some of its Medical Uses”, 1789) it had been suspected that digitalis acted as a substitute for an endogenous mammalian digitalis compound. The search for such a compound, however, was futile for a long time because of methodological limitations. Mammalian endogenous inhibitors of Na^+^, K^+^ adenosine triphosphatase (ATPase) were found decades ago, but only recent methodological breakthroughs have permitted us to identify cardenoloids (ouabain, digoxin) and bufadienolides (marinobufagenin), i.e. substances which have marked effects on blood pressure, natriuresis and other functions [38]. Marinobufagenin, which was originally found in the skin of toads, is natriuretic [62]. Its blood concentration varies with changes in environmental salinity [63]; it causes vasoconstriction in the mammalian organism [64], and its concentration increases after volume expansion [65] as well as in pathological states of volume expansion, e.g. uremia and pre-eclampsia [64, 66].

**Fig. 3:**
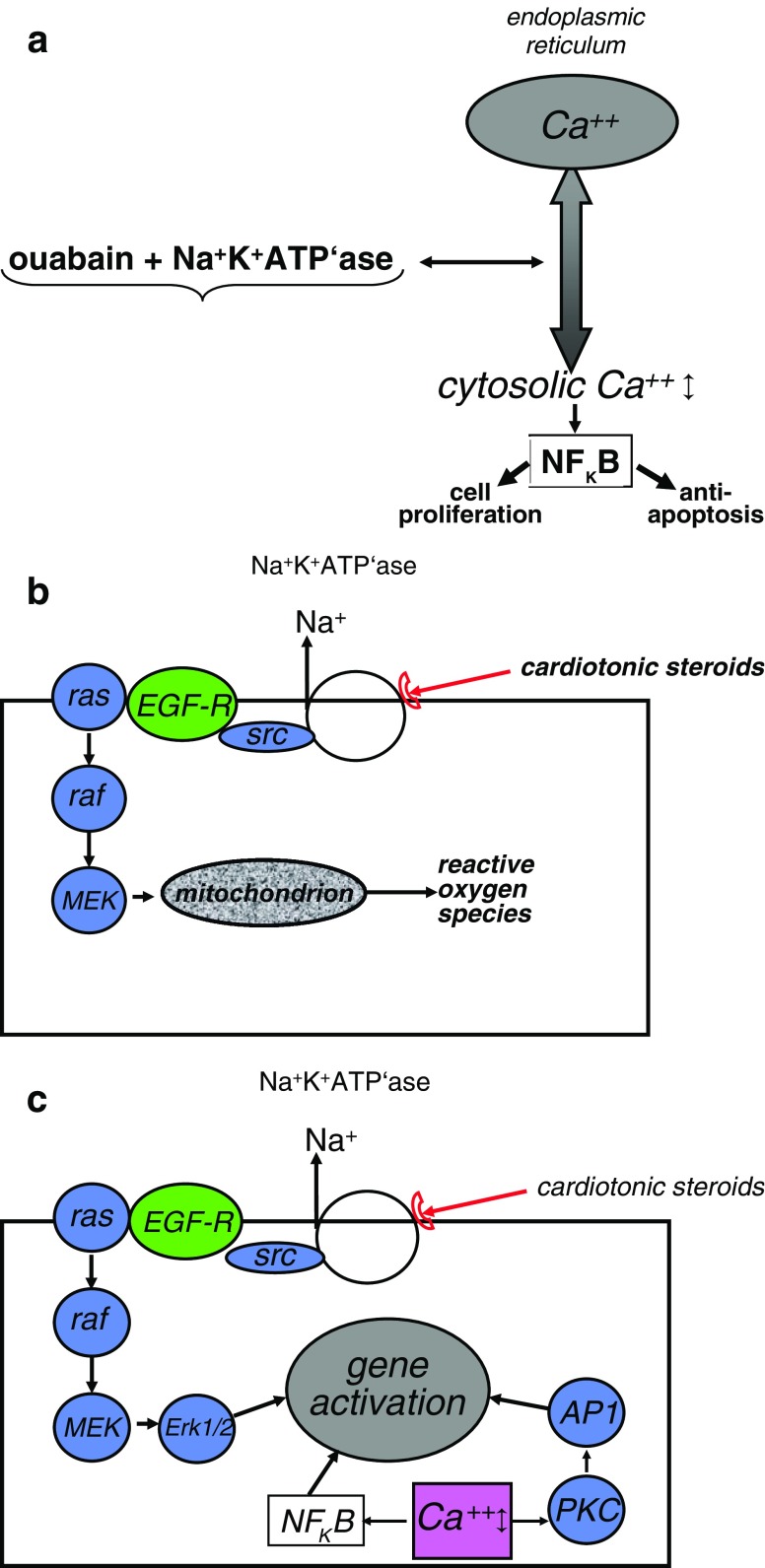
Signaling pathways of cardiotonic steroids **a** Activation of NFκB via slow Ca^++^ oscillations following modification of Ca^++^ release from endoplasmic reticulum. **b** Activation of mitochondrial ROS via *src* and EGF-R. **c** Activation of nuclear gene programs via *scr* and EGF-R. *PKC* protein kinase C

The ideas concerning the mode of action of these compounds have recently undergone a transformation. In the past it had been assumed that these inhibitors of sodium potassium ATPase are inotropic in the heart because of an increase in intracellular calcium (Ca^++^) resulting from accumulation of intracellular sodium and backward running of the Na^+^Ca^++^ exchanger. More recently, however, it has been found that they act as signal transducers at nanomolar or sub-nanomolar concentrations which do not inhibit the Na^+^,K^+^-ATPase: they bind to the extracellular domain of the α-1 chain of Na^+^, K^+^-ATPase, cause slow Ca^++^ oscillations by interaction with the endoplasmic reticulum (Fig. 3a), generate reactive oxygen species by acting on the mitochondria (via src, epidermal growth factor receptor (EGF-R), ras, raf, MEK) (Fig. 3b) and activate nuclear gene programs [67], mainly as the result of the activation of nuclear factor-kappa B (NFκB) by the slow calcium oscillations (Fig. 3c).

The signaling is mediated by interaction of the N-terminus of Na^+^,K^+^-ATPase with cardiotonic steroids, creating a so-called signalosome. Such signaling influences cell proliferation (apoptosis) and cell–cell contact (cell migration)—this has led to the development of anti-cancer agents. In the kidney, the effect of inhibiting apoptosis and stimulating cell proliferation via activating NFκB has been well documented [68]. Such signaling also modulates natriuresis and blood pressure—this even led to the development of novel anti-hypertensive substances [69].

With respect to uremia, it is of considerable interest that, in subtotally nephrectomized rats, endogenous marinobufagenin concentrations are increased [70]. If similar concentrations were achieved by infusing marinobufagenin in rats subjected to sham operation, cardiac hypertrophy with thickening of the left ventricular wall, cardiac fibrosis, diastolic malfunction and signaling via Src/ERK were observed. All these adverse effects could be prevented by pre-immunization, yielding neutralizing antibodies against marinobufagenin. In dialysis patients a correlation was found between endogenous ouabain by radio-immunosorbent assay (RIA) and mass spectrometry and left ventricular (LV) mass, LV volume and eccentric LV hypertrophy [71].

It is likely that, apart from in the heart, cardiotonic steroids play a role in the genesis of other types of uremia-induced target organ damage as well.

## Conclusion

There is strong evidence that salt intake plays an important role in the genesis of hypertension and target organ damage. Both high and low sodium intake cause adverse effects. The average salt intake of healthy children and adults exceeds, by far, the recommendations of current guidelines. The sodium content of most commercially available food items is too high, and this accounts for nearly three-quarters of salt intake. The most important aspect of strategies to reduce salt intake is an effort to make the food industry reduce nutrient salt content. Especially in renal patients, diuretics are usually needed in addition to reduction of dietary salt intake. The fascinating insights into novel pathomechanisms underlying salt-induced target organ damage, e.g. cardiotonic steroids, may ultimately yield new therapeutic interventions.
